# Pediatric Patient With a Giant Vertebrobasilar Dissecting Aneurysm Successfully Treated With Three Pipeline Embolization Devices

**DOI:** 10.3389/fneur.2020.00633

**Published:** 2020-07-03

**Authors:** Luqiong Jia, Jiejun Wang, Longhui Zhang, Yunfeng Zhang, Wei You, Xinjian Yang, Ming Lv

**Affiliations:** ^1^Department of Interventional Neuroradiology, Beijing Neurosurgical Institute and Beijing Tian Tan Hospital, Capital Medical University, Beijing, China; ^2^Department of Imaging and Nuclear Medicine, Baoding No.1 Central Hospital, Baoding, China

**Keywords:** pediatric patient, giant, vertebrobasilar dissecting aneurysm, three pipeline, flow diverter (FD)

## Abstract

Pediatric intracranial dissecting aneurysms are rare (1), and treating this type of aneurysm in the vertebrobasilar circulation is more difficult. As an off-label application, pipeline embolization devices (PEDs) for posterior circulation dissecting aneurysms are reported to have good therapeutic effect (2). However, studies have found that PEDs for large or giant vertebrobasilar dissecting aneurysms have a poor effect and are associated with disastrous consequences for patients (3). PEDs are feasible for vertebrobasilar dissecting aneurysms (4); however, few reports discuss using PEDs to span the entire segment of the basilar artery. Because there are more perforating arteries in the basilar artery, it is more prudent to use PEDs in this artery. We report a case of a pediatric patient with a giant vertebrobasilar dissecting aneurysm successfully treated with three PEDs combined with right vertebral artery occlusion, without complications. The patient's headache symptoms resolved fully 3 months after the procedure, and the aneurysm was completely healed and excellent reconstruction of the left vertebral artery was seen 4 months post-procedure, using digital subtraction angiography.

## Report of a Case

**Figure 1:**
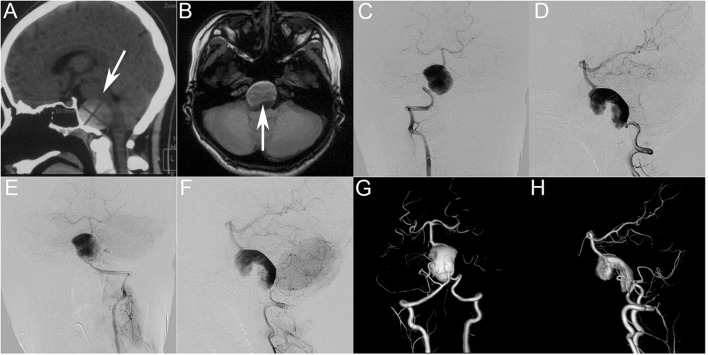
**(A,B)** Preoperative computed tomography **(A)** and magnetic resonance imaging **(B)** showing a giant intracranial mass effect (white arrow) in the basal ganglia region. Preoperative anteroposterior angiograms of the right vertebral artery **(C,D)** and left vertebral artery **(E,F)** showing a giant dissecting aneurysm in the vertebrobasilar artery. **(G,H)** Three-dimensional reconstruction showing that the dissecting aneurysm straddles bilateral vertebral arteries and that the anterior inferior cerebellar artery arises from the aneurysm.

A pediatric patient presented with a persistent headache after recovering from a cold. Computed tomography and magnetic resonance angiography (Figure 1) at another hospital revealed a giant dissecting aneurysm in the vertebrobasilar artery. Digital subtraction angiography (DSA) confirmed that a giant dissecting aneurysm was located in the vertebrobasilar artery. Three-dimensional reconstruction showed that the dissecting aneurysm straddled bilateral vertebral arteries and that the anterior inferior cerebellar artery arose from the aneurysm. After a comprehensive discussion between five neurointerventional experts and three neurosurgical experts, we decided to treat the right vertebral artery (RVA) occlusion and to use pipeline embolization devices (PEDs) to reconstruct the left vertebral artery (LVA) and basilar artery to embolize the aneurysm.

**Figure 2:**
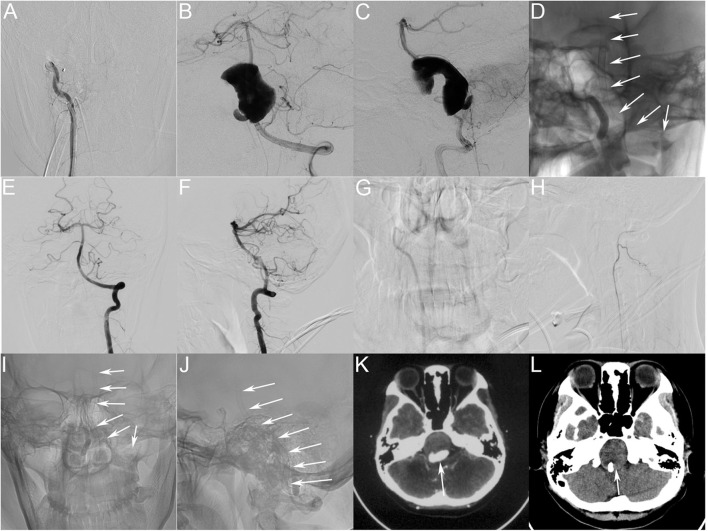
**(A)** Immediately post-operative angiograms showing the complete occlusion of the RVA. **(B,C)** Immediately post-operative angiograms showing the reconstruction of the vertebrobasilar artery and visible contrast stasis in the lumen of the aneurysm. **(D)** Unsubtracted view showing good shape of the three PEDs (white arrow). **(E,F)** Four-month post-treatment DSA of the LVA showing complete occlusion of the aneurysm and reconstruction of the LVA and basilar artery. **(G,H)** Four-month post-treatment DSA of the RVA showing that the occluded RVA is thinner, and the posterior inferior cerebellar artery has good blood flow. **(I,J)** Four-month post-treatment angiograms (unsubtracted view) showing good shape of the PEDs (white arrow). **(K,L)** Four-month post-treatment computed tomographic image **(L)**. Compared with the preoperative image **(K)** the post-operative image shows decreased aneurysm size (white arrow) and increased space around the brainstem. PED, pipeline embolization device; RVA, right vertebral artery; DSA, digital subtraction angiography; LVA, left vertebral artery.

The patient was premedicated with a dual antiplatelet regimen [75 mg clopidogrel and 100 mg of acetylsalicylic acid (ASA) daily] for 5 days prior to treatment. All endovascular treatments were performed under general anesthesia by experienced neurointerventionists. After canalizing the femoral artery with a 6-F arterial sheath adjusted to the proper working angle, using the predesigned road map, we placed a 5 F guiding catheter (Codman; Raynham, MA, USA) in the distal V4 segment of the RVA. We occluded the RVA with a detachable Gold balloon #2 (Balt, Montmorency, France), and DSA confirmed complete occlusion of the RVA (Figure 2). Next, we guided a 6-F Navien Intracranial Support Catheter (Covidien Vascular Therapies, Mansfield, MA) to the desired LVA position. Using the road map, we used a Synchro-14 (Stryker, Kalamazoo, MI) microguidewire to advance a Marksman (EV3; Irvine, CA) stent catheter to the middle of the basilar artery, after which, we withdrew the microguidewire. With the assistance of multiple projection angles, we released the PED (3.25 × 35 mm) after the PED reached the desired position. However, we found that the stent was severely shortened and had a tendency to fall into the aneurysm sac after its release. Therefore, following urgent discussion in the expert group, we decided to bridge two more stents: one at the distal end and another at the proximal end of the aneurysm. We navigated the Marksman stent catheter to the apex of the basilar artery and released another PED, resulting in an overlapping bridge of ~5 mm. We moved the Marksman to ~7 mm from the distal end of the first PED and successfully released the third PED. Intraoperative DSA showed that the three PEDs were positioned well, and the parent artery was patent with contrast stasis in the lumen of the aneurysm. We then withdrew the Marksman stent catheter. The Synchro-14 microguidewire forms a loop with the PEDs supported by an Echelon-10 microcatheter (ev3 Endovascular; Plymouth, MN). Immediately post-operative DSA of the LVA showed that the PEDs adhered well to the wall and confirmed the reconstruction of the parent vessel and contrast stasis. We then removed all catheters and completed the operation. During the procedure, the patient received intravenous heparin to maintain an activated clotting time of ~250–300 s.

After recovering from anesthesia, the patient was conscious and could follow our instructions. On the second day after the operation, the patient had recovered well and was discharged from hospital without complications. She complained of residual headache at discharge, and her modified Rankin scale score was 1. Post-operatively, dual antiplatelet therapy (75 mg clopidogrel and 100 mg of ASA daily) was continued for 6 months. Clopidogrel was discontinued after 3 months, and ASA was continued for life.

We follow this patient every other month by telephone. The patient stated that the headache had improved ~20 days after the operation, and completely resolved 3 months after the operation. Angiographic follow-up data were obtained 4 months after the endovascular treatment. DSA demonstrated excellent reconstruction of the LVA and confirmed complete healing of the aneurysm.

## Discussion

We described a patient with a giant vertebrobasilar dissecting aneurysm, which involved very complicated treatment. The patient had a persistent headache, which we considered was secondary to the mass effect of the aneurysm. Therefore, we did not adopt a traditional stent-assisted coil treatment strategy because filling the aneurysm sac with the coil would not have relieved the mass effect caused by the aneurysm. In addition, traditional stent-assisted coiling is associated with a high aneurysm recurrence rate. We chose to occlude the RVA because the LVA is straighter than the RVA, and the increased tortuosity in the RVA cannot ensures that the PED has good adhesion to the wall. In this patient, our plan was to place only one PED; however, the first PED shortened severely after placement and had a tendency to fall into the aneurysm sac. Therefore, we had to bridge two more PEDs: one at the proximal end and another at the distal end of the first PED. There is no doubt that PEDs can cover the entire basilar artery, which is the concern in patients at risk of perforation; however, using PEDs in the basilar artery has been reported rarely. The use of stents to treat patients with basilar artery apex aneurysms was reported by Adam et al. one patient (6.3%) died secondary to posterior cerebral artery and cerebellar strokes as well as subarachnoid hemorrhage after the placement of a flow diverter. Additionally, the authors found that incomplete occlusion occurred more commonly in patients treated with PEDs alone compared with those undergoing concurrent coiling, at the 6-month angiographic follow-up (5).

Our patient experienced no complications after endovascular treatment. DSA showed complete occlusion of the aneurysm 4 months after the endovascular treatment, and computed tomography showed that the mass effect had decreased. The findings in our patient show that PED bridging is feasible for the treatment of pediatric giant vertebrobasilar dissecting aneurysms, but our results require further investigation in clinical trials.

## Data Availability Statement

The raw data supporting the conclusions of this article will be made available by the authors, without undue reservation, to any qualified researcher.

## Ethics Statement

The studies involving human participants were reviewed and approved by Beijing Tiantan ethics committee. Written informed consent to participate in this study was provided by the participants' legal guardian/next of kin.

## Author's Note

This work was originated from Beijing Neurosurgical Institute and Beijing Tian Tan Hospital, south fourth ring road west 119, Fengtai District, Beijing.

## Author Contributions

LJ wrote the first draft of the manuscript. JW acquired the data. LZ and YZ analyzed and interpreted the data. WY edited the figure of the article. XY and ML conceived and designed the research. All authors contributed to the article and approved the submitted version.

## Conflict of Interest

The authors declare that the research was conducted in the absence of any commercial or financial relationships that could be construed as a potential conflict of interest. The reviewer YZ, declared a shared affiliation, though no other collaboration, with the authors to the handling editor.
